# Anthropogenic impacts in protected areas: assessing the efficiency of conservation efforts using Mediterranean ant communities

**DOI:** 10.7717/peerj.2773

**Published:** 2016-12-14

**Authors:** Elena Angulo, Raphaël Boulay, Francisca Ruano, Alberto Tinaut, Xim Cerdá

**Affiliations:** 1 Estación Biológica de Doñana, CSIC, Sevilla, Spain; 2 Institut de Recherches sur la Biologie de l’Insecte, CNRS UMR 7261, Université François Rabelais de Tours, Tours, France; 3 Departamento de Zoología, Universidad de Granada, Granada, Spain

**Keywords:** Ants, Biodiversity, Biotic homogenization, Disturbance, Management, Endemism, Species richness, Spain, Andalusia, Ants

## Abstract

In countries with high levels of urbanization, protected areas are often subject to human disturbance. In addition to dealing with fragmentation, land managers also have to confront the loss of characteristic ecosystems due to biotic homogenization, which is the increasing similarity of species assemblages among geographically separate regions. Using ants as a model system, we explored whether anthropogenic factors negatively affect biodiversity of protected areas of a regional network. We first analysed the effect of fragmentation and human activity on ant biodiversity within protected areas. Secondly, we tested whether homogenization could occur among protected areas. We sampled 79 plots in the most common habitats of 32 protected areas in southern Spain and calculated ant community richness and diversity indices, endemic richness, and Bray–Curtis similarity indices (between pairs of plots). We related these indices with patch fragmentation and human disturbance variables, taking into account environmental, spatial and landscape covariates. We used ANOSIM to test for differences between similarity indices, specifically among levels of anthropogenic disturbance. Species richness was positively correlated with the distance from the border of the protected areas and the number of endemic species was negatively correlated with the degree of fragmentation. Ant communities were similar within each protected area but differed across regions. Human disturbance was not correlated with community similarity among sampling points. Our approach suggests how the ability of European protected areas to sustain biodiversity is limited because they remain susceptible to anthropogenic impacts. Although ant communities maintained their biological distinctiveness, we reveal how fragmentation within protected areas is important for community richness and endemism maintenance.

## Introduction

Establishing and managing protected areas (PAs) are core biodiversity conservation strategies (Lovejoy, 2006). Over the last decades, the creation of new PAs has become a requirement imposed on the signatories of the most important conservation conventions in the world, including the Convention on Biological Diversity (Secretariat of the Convention on Biological Diversity, 2008), the European Union Biodiversity Action Plan (EC, 2006), and the National Ecological Observatory Network (Keller et al., 2008). At the same time, research focusing on the design, management, and ecological integrity of PAs has been conducted (Gaston et al., 2008; Andrello et al., 2015; Bartonova et al., 2016). Although the number of studies that have tested the effectiveness of PAs in sustaining biodiversity is still limited (Rodrigues et al., 2004; Geldmann et al., 2013), some recent work suggests that PAs may not always allow a satisfactory level of conservation to be reached, at least in the case of some taxa (Gaston et al., 2008; Rayner et al., 2014; Jenkins et al., 2015a; but see Brown et al., 2015). More importantly, the assessment of performance of already established PAs relies on how successfully biodiversity features are maintained within PAs, buffering them from external pressures (Gaston et al., 2008; Jenkins et al., 2015b). Thus, it is important to understand how effectively PAs networks are performing for biodiversity conservation and the factors that best predict these outcomes.

The ability of PAs to sustain biodiversity is frequently severely limited because they often remain susceptible to anthropogenic impacts. First, biodiversity in PAs may be impacted by human exploitation of ecosystem services, such as tourism, recreation, or the sustainable extraction of resources. For example, in increasingly urbanized and densely populated European countries, PAs are often located close to cities (McDonald et al., 2009). This placement pattern tends to encourage people to visit and use PAs, which may, in turn, increase habitat fragmentation, for example due to urbanization or agriculture expansion (Martinuzzi et al., 2015). As Gaston et al. (2008) argued, the importance of PAs networking has become more critical as the connectivity between human populations tends to increase and intervening areas become less hospitable to species. As has been demonstrated by long-term field-based fragmentation experiments, the effects of habitat fragmentation are strong and markedly consistent across a diverse array of terrestrial ecosystems on five continents (Haddad, Brudvig & Clobert, 2015; Wilson et al., 2016). The Millennium Ecosystem Assessment stressed that, in recent decades, European ecosystems have suffered more human-induced fragmentation than ecosystems on any other continent (MEA, 2005; EC, 2006). Thus, by detecting the degree of fragmentation present in networks of established PAs, we may be able to obtain crucial information that will allow PAs to be managed in such a way as to enhance conservation.

Second, biotic homogenization describes the ecological processes by which formerly disparate biotas lose biological distinctiveness at any level of organization (Olden & Rooney, 2006). This phenomenon is often caused by an increase in the abundance/incidence of invasive or common species and a consecutive decrease in abundance/incidence of endemic species, which tends to enhance the similarity of species assemblages among geographically separate regions. In Europe, the close proximity of PAs to farmlands and cities means that many of the species contributing to overall biodiversity originate from lands outside the PAs (MEA, 2005). Biotic homogenization may also be generated by environmental changes, such as changes in land use, which promote the geographic expansion of some species and result in the shrinking distributions of others (Florencio et al., 2013; Florencio et al., 2015; Harrison et al., 2014; Solar et al., 2015). However, there is little information available regarding the changes in compositional similarity among local communities that have taken place within PA networks, especially for animals.

More than half of the Iberian Peninsula is a Mediterranean biodiversity hotspot (Médail & Quézel, 1999; Myers et al., 2000). The Andalusia region, which is located in the southern Iberian Peninsula (Spain), contains 80 PAs, including two national parks. These PAs encompass diverse ecosystems and landscapes, including coastal zones, high mountains, deserts, and mesic forests. Overall, they represent 30.5% of the total surface area of Andalusia, which is more than twice the European average (13.7%). Furthermore, PAs in the Iberian Peninsula have been shown to contain a large representation of regional plant and animal species diversity (>73%; Araújo, Lobo & Moreno, 2007). However, in spite of the heavy reliance that conservation places on PAs networks, there is no study identifying the possible shortfalls in the conservation performance of already established PAs (Gaston et al., 2008).

The aim of the current study was to analyse the effects of anthropogenic impacts on biodiversity within and among Andalusia’s PAs. We used ants as model organisms as they are good ecological indicators in most terrestrial ecosystems (Andersen & Majer, 2004; Sauberer et al., 2004); they are also easy to sample and have high species and functional richness (King, Andersen & Cutter, 1998; Underwood & Fisher, 2006; Sanford, Manley & Murphy, 2009). First, we investigated the relationship between ant diversity and anthropogenic disturbance in Andalusia’s PAs; we used several measures of disturbance, including habitat fragmentation, and accounted for covariates such as climate and habitat type. Specifically, we examined anthropogenic factors that could negatively affect biodiversity within PAs, such as distance to PA borders or to human elements (houses, roads, trails), vegetation patch sizes, presence of human activity (garbage, crops). Secondly, we explored the degree to which ant communities demonstrated biotic homogenization across Andalusia’s PAs. If local ant communities were relatively intact, then similarity between communities should decrease with increasing distance. Alternatively, similarity could increase at high disturbance levels. In doing so, we assess the vulnerability of biodiversity within and among protected areas and identify the factors affecting biodiversity conservation in this protected area network.

**Figure 1:**
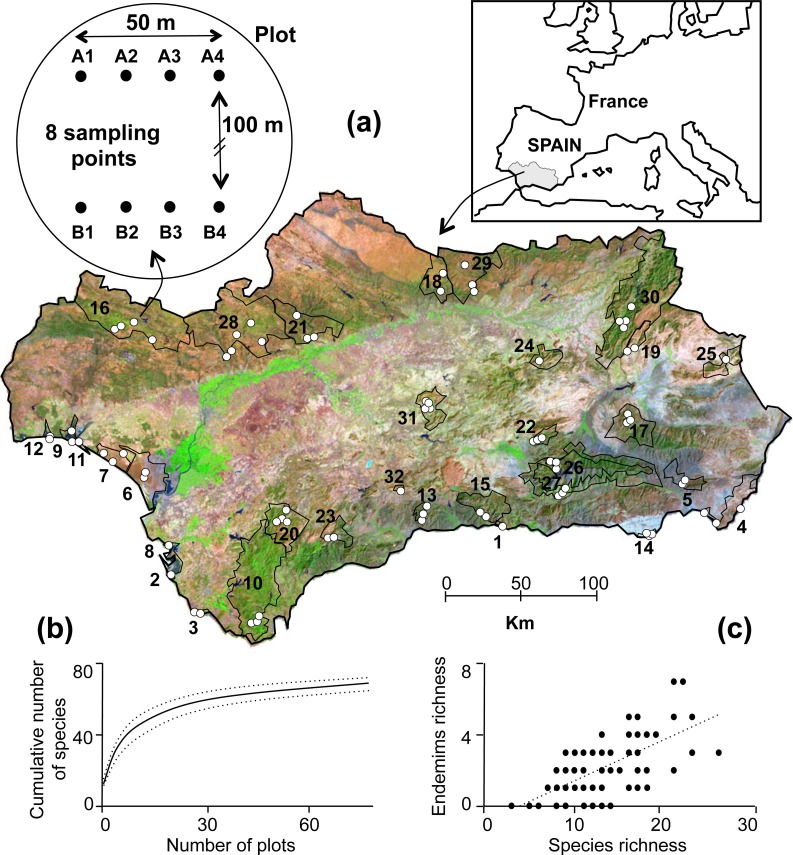
(A) Study area and sampling protocol. Seventy-nine plots across 32 protected areas in Andalusia (southern Spain) were sampled, for a combined total of 607 sampling points. See Table S1 for the codes of the protected areas. (B) Cumulative number of species sampled in this study across the 79 plots in South Spain (dotted lines represent 95% confidence intervals). (C) Relationship between endemism and species richness (*R*^2^ = 0.39, *p* < 0.001, *N* = 79).

## Material and Methods

### Study areas and ant sampling procedures

In the summers of 2005, 2006, and 2007, we sampled ant communities in 32 of Andalusia’s PAs, including two national parks, 22 natural parks, and eight areas falling in other categories of protection (Fig. 1A; Table S1). The weather in summer is very homogenous in Mediterranean habitats, which warrants that all the sampling was done in similar conditions. In each PA, taking into account its size and main habitats, we selected one to five plots in different habitats that were representative of the area. In each plot, we established two parallel 50-m-long transects 100 m apart. We determined their position using a standard GPS unit (Garmin eTrex^®^10). There were four sampling points along each transect, and seven pitfall traps (200-ml plastic cups 2/3 filled with soapy water) at each sampling point (Fig. 1A); the traps were open to collect ground foraging invertebrates for 24 h. This short time is sufficient to capture the most abundant and ecologically relevant species to compare their diversity and richness among sites and in relation to anthropogenic factors; moreover the relatively short sampling time was chosen to avoid to capturing non-targeted endangered species (such as small lizards, toadlets or pygmy shrews). Regional Government of Andalusia (Consejería de Medio Ambiente) issued permits for the fieldwork in PAs and some field facilities were provided by ICTS-RBD. We pooled the contents of the seven pitfall traps for a given sampling point and kept in 70% alcohol until species could be identified in the laboratory. We excluded four sampling points from the study because more than four of their pitfall traps disappeared during the sampling period. In the other 25 sampling points, in which four or less pitfall traps had disappeared, we estimated ant abundance extrapolating from the number of active traps. Overall, we used data from 607 sampling points spread across 79 plots.

### Diversity indices

For each plot, we calculated the following diversity indices: (1) observed species richness (*S* = the total number of species sampled per plot); (2) estimated species richness (Chao); (3) the Shannon diversity index (*H*), which accounts for both the number of species and their relative abundance; (4) the proportion of Andalusian endemic species (*Ea*); and (5) the proportion of Iberian endemic species (*Ei*). The classification in Andalusian and Iberian endemic species was done using available distribution maps (www.hormigas.org and www.fauna-eu.org). We calculated Chao and *H* using a sample-based randomization procedure in EstimateS that employed abundance data from the eight sampling points per plot (Colwell, 2009). When the bias-corrected Chao was associated with a coefficient of variation higher than 0.5 for the abundance distribution, we used the classic Chao 1 estimator, as recommended by Colwell (2009). We also converted Shannon diversity index to true diversity or effective number of species (Jost, 2006). We calculated *Ea* and *Ei* using the observed number of ant species per plot. Finally, we represented the values of the conservation indices in maps, constructed by interpolation from data plots, using ArcGis 9.3 (ESRI) and the interpolation tool of the Spatial Analist package (ESRI).

### Similarity indices

We log transformed species abundance, or the number of trapped ants of each species in each plot, to calculate the Bray–Curtis similarity index (Sorensen quantitative index) between pairs of plots using PRIMER v.6 (Clarke & Gorley, 2006). The log transformation is recommended to deal with skewed data such as ant abundance, which have a wide variability. The Bray–Curtis similarity index uses pairwise comparisons to give an indication of the proportion of species shared between ant assemblages. We excluded the plot in which the only species present was the invasive Argentine ant (*Linepithema humile*).

### Anthropogenic and environmental variables

#### Spatial and environmental variables

We obtained plot latitude, longitude, and elevation (LAT, LONG, and ELE) using the GPS coordinates. For each plot, we obtained estimates of seven relevant bioclimatic variables (Table S2) from the WorldClim database (www.worldclim.org, Hijmans et al., 2005); these variables described average climatic conditions for the period 1950–2000. Using principal components analysis, they were reduced down to two uncorrelated principal components that accounted for 79.4% of the original variation (CLIMCOMP1, CLIMCOMP2, Table 1).

**Table 1:** Summary of the variables included in the statistical models used to analyze their effect on ant diversity across Andalusia’s PAs.

Variable name		Variable description
Spatial variables		
	LAT	Latitude
	LONG	Longitude
	ELE	Elevation
Environmental variables		
	CLIMCOMP1	Gradient of mild to extreme climates
	CLIMCOMP2	Gradient of Mediterraneity: from humid and cool areas to dry and warm areas (following Carvalho et al., 2011)
	SOILCOMP1	Soil richness in terms of organic material and potassium
	SOILCOMP2	Soil granulometry, high concentration of fine sand, and low concentration of phosphorus
	SOILCOMP3	Largely soil pH
Habitat variables		
	VEGTYP	Vegetation type
	VEGCOV	Vegetation cover, mean % of cover
	SEAD	Distance to the sea
	FRESHD	Distance to a source of fresh water (e.g., streams and rivers)
	GEOLAND	Geological landscape type (northern and southern mountain chains, and littoral)
Fragmentation (of the vegetation patch in which the plot is located)		
	PATBOR	Distance to the edge of the vegetation patch
	PATAR	Area of the vegetation patch
	PERAR	Perimeter-to-area of the vegetation patch
Anthropogenic variables		
	PLOTBOR	Distance to the boundary of the protected area
	ANTDI	Index of anthropogenic disturbance, which takes into account the presence of garbage, soil manipulation, trails, roads, and/or human construction within 500 m

We sampled one kilogram of soil at the start point of each transect which was later analyzed at the IRNAS (Instituto de Recursos Naturales y Agrobiología de Sevilla, CSIC, Seville, Spain). We measured seven soil composition variables for each plot (Table S2). These variables were then reduced down to three principal components, which accounted for 80.4% of the original variation (SOILCOMP1, SOILCOMP2, SOILCOMP3; Table 1).

#### Habitat variables

We placed plots in the following vegetation type (VEGTYP) categories: pine forest, Holm oak (*Quercus ilex*) forest, mixed forest, olive tree plantation, scrubland, dune, or pasture. In addition, we assessed the mean vegetation cover (VEGCOV) on each plot using four photographs; we placed a digital camera (Fujifilm FinePix F50fd) face up on the ground at the start point and the end point of each transect (=four photographs per plot). The photographs were taken with identical angular field of view focusing at infinity. We converted the photographs to black and white images using Adobe Photoshop^®^. We estimated plot vegetation cover by calculating the average percentage of black pixels found on the four pictures for each plot.

We also calculated the Euclidean distance in meters to the sea (SEAD) and to a source of fresh water (FRESHD), such as a river or a lake, for each plot; we did this using ArcGis 9.3 (ESRI), available georeferenced information (DEA100, 2009), and the tool package ET Geo Wizards 10.0 (ESRI). We then categorized PAs based on the three main geological landscapes (GEOLAND) established by the Center of Landscape and Territory Studies (http://www.paisajeyterritorio.es/) northern mountain chain, southern mountain chain, and littoral.

#### Fragmentation

Using orthophotographs from Andalusia’s GIS site (http://www.juntadeandalucia.es/institutodeestadisticaycartografia/prodCartografia/ortofotografias/orto09.htm) and ArcGis 10 (ESRI) we defined patches of continuous vegetation around each plot. We delimited patch boundaries by any abrupt change in habitat type or the presence of roads, trails, or firewalls, and within a given patch, areas characterized by a different type of habitat were excluded. We then measured the minimum distance between the plot centroid and the patch border (PATBOR); the total patch area (PATAR); and the perimeter-to-area ratio (PERAR). The latter provides a measure of fragmentation, where a lower perimeter-to-area ratio means that patches are more circular and thus less fragmented and less impacted by humans (e.g., Collinge & Palmer, 2002).

#### Anthropogenic disturbance estimates

We estimated the degree of anthropogenic disturbance in the plots by measuring the minimum distance to the closest PA’s border (PLOTBOR), considering PA’s borders as a source of disturbance. In addition, we developed an anthropogenic disturbance index (ANTDI) that ranged from 0 (no apparent disturbance) to 5 (high levels of disturbance). In this index, we assigned a point for each of the following signs of human disturbance (assessed in the field and verified using orthophotographs): the presence of garbage, crops or evidence of the soil having been manipulated (e.g., ploughing), and the presence of a trail, a road, or construction within 500 m of the GPS position of the plot. A score of 0 therefore meant no signs of disturbance were present, whereas a score of 5 meant that they were all present.

### Data analysis

First, we analyzed pairwise correlations among the five diversity indices using linear regressions (GRM, STATISTICA 8.0, StatSoft Inc., 2007). Thus, eight correlations were carried out in order to remove any highly correlated index.

Second, we examined the relationship between the diversity indices and the anthropogenic variables and the covariates (Table 1) using general linear regression models (Genmod Proc in SAS; SAS, 9.1.3; SAS Institute, Cary, NC, USA). We employed a backward stepwise model selection procedure and a distribution of errors that minimized model deviance (Herrera, 2000); that is, for each index, we carried out a regression model with the most adequate distribution of errors and the variables in Table 1. PAs were included as a repeated subject because there was more than one plot per PA. In addition, we performed a spatial autocorrelation test (using the Proc Variogram in SAS; SAS, 9.1.3; SAS Institute, Cary, NC, USA) which provides Moral I and Geary *c* statistics. The richness and Iberian endemism indices showed significant spatial autocorrelation while the Shannon and the Andalusian endemisms indices did not. In any case, we added to the model latitude and longitude, as well as their interaction, to better account for spatial autocorrelation.

Third, we analyzed ant community similarity among groups of plots using ANOSIM implemented in PRIMER v.6 (Clarke & Gorley, 2006). ANOSIM calculates a statistic called global *R*, which varies between −1 and +1. A higher global *R* value denotes greater differences among than within groups of plots. We conducted four sets of analyses in which groups were successively defined as plots (1) that belonged to the same PAs; (2) that had the same vegetation type; (3) that had the same geological landscape type; and (4) that were characterized by the same level of anthropogenic disturbance. Because there was only one plot in each of the extreme categories of anthropogenic disturbance (0 and 5), we merged categories 0 and 1 and categories 4 and 5. Similarity was graphically displayed using a non-metric multidimensional scaling (nMDS) plot, and by estimating the mean (SD) Bray–Curtis similarity indices for pairs of communities belonging to the same group.

## Results

### Ant diversity in Andalusia’s protected areas

**Figure 2:**
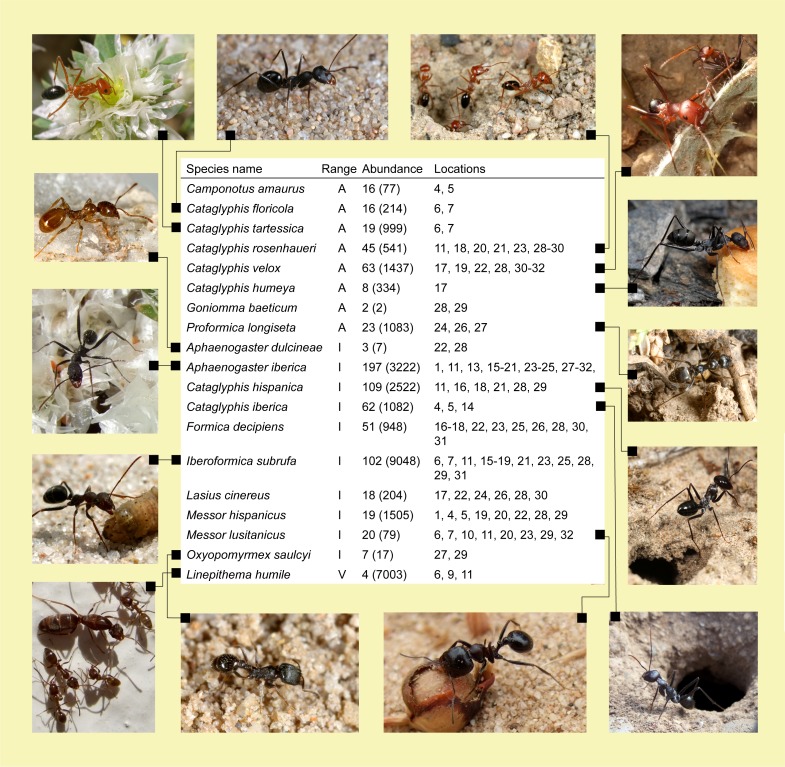
Endemic and invasive species found in protected areas of south Spain. The range of distribution is categorized in Iberian (I) or Andalusian (A) endemisms, or invasive species (V). The abundance corresponds to the number of plots with presence of each species from a total of 607, and in brackets is the total number of ants. Location numbers correspond to protected areas in Fig. 1.

**Figure 3:**
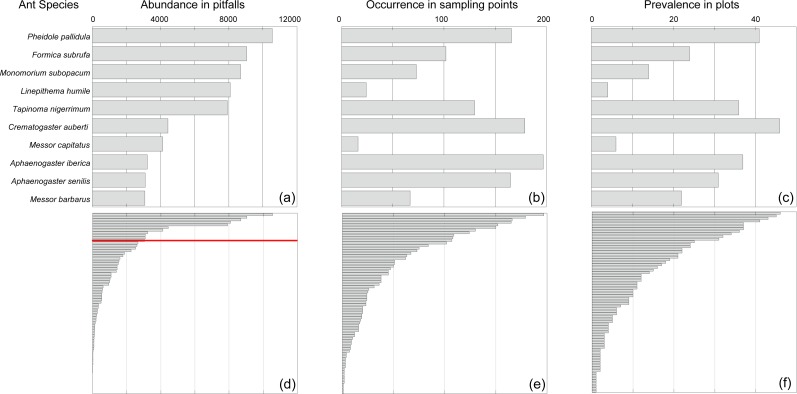
Abundance in pitfalls (A), occurrence in sampling points (B) and prevalence across plots (C) of the tenth more abundant species (above the red line in D) and of the remaining species (D–F).

We sampled a total of 100,139 ants belonging to 70 species and 23 genera across 79 plots distributed throughout the 32 PAs studied (Table S1). On average (SD), we captured 11.5 (4.6) species per plot (range: 1–24). The plot with the lowest species richness (*S* = 1) was occupied by the Argentine ant (*Linepithema humile*); the only invasive species sampled during this study. Observed species richness (*S*) was close to predicted species richness (Chao (SD) = 13.2 (6.7)). The nearly asymptotic shape of the species accumulation curve (Fig. 1B) suggested that our procedure allowed us to sample the majority of the species present in the region (Colwell & Coddington, 1994). Out of the 70 species sampled, eight and 10 are endemic to Andalusia and the Iberian Peninsula, respectively (Fig. 2). Seven of these endemic species belong to the genus *Cataglyphis* (Subfamily Formicinae), which is adapted to arid and hot environments. We found a maximum of two Andalusian and five Iberian endemic species per plot. Taking into account abundance, occurrence in sampling points and prevalence across plots the most abundant species were *Pheidole pallidula*, *Tapinoma nigerrimum*, *Crematogaster auberti*, and two *Aphaenogaster* species (Fig. 3). The sole invasive species, *L. humile*, represented 8.1% (8,081 individuals) of all individuals captured but it was present in only 24 sampling points and 4 plots). Nine species occurred only rarely (i.e., they were present in 1 or 2 sampling points of 1 or 2 plots and less than 10 individuals were captured): *Temnothorax prope naeviventris*, *Goniomma baeticum*, *Lasius brunneus*, *Strongylognathus testaceus*, *Monomorium algiricum*, *Goniomma hispanicum*, *Aphaenogaster dulcineae*, *Camponotus ruber*, and *Lasius myops* (Table S3).

Observed species richness (*S*) was positively correlated with the total number of endemic species—the sum of Andalusian and Iberian endemic species (*R*^2^ = 0.39, *p* < 0.001, *N* = 79, Fig. 1C). The mean (SD) Shannon diversity index was *H* = 1.35 (0.5); it ranged from 0 to 2.2. This means an effective number of species (or true diversity) of 4.31 (1.91) ranging from 1 to 9.2. *H* was positively correlated with S (*R*^2^ = 0.45, *p* < 0.001, *N* = 79) and Chao (*R*^2^ = 0.36, *p* < 0.001, *N* = 79), but not with *Ea* or *Ei* (*p* > 0.1). None of the previous correlations were sufficiently strong to remove an index; so further analyses were done with all indices.

### Anthropogenic and environmental determinants of ant diversity within protected areas

**Figure 4:**
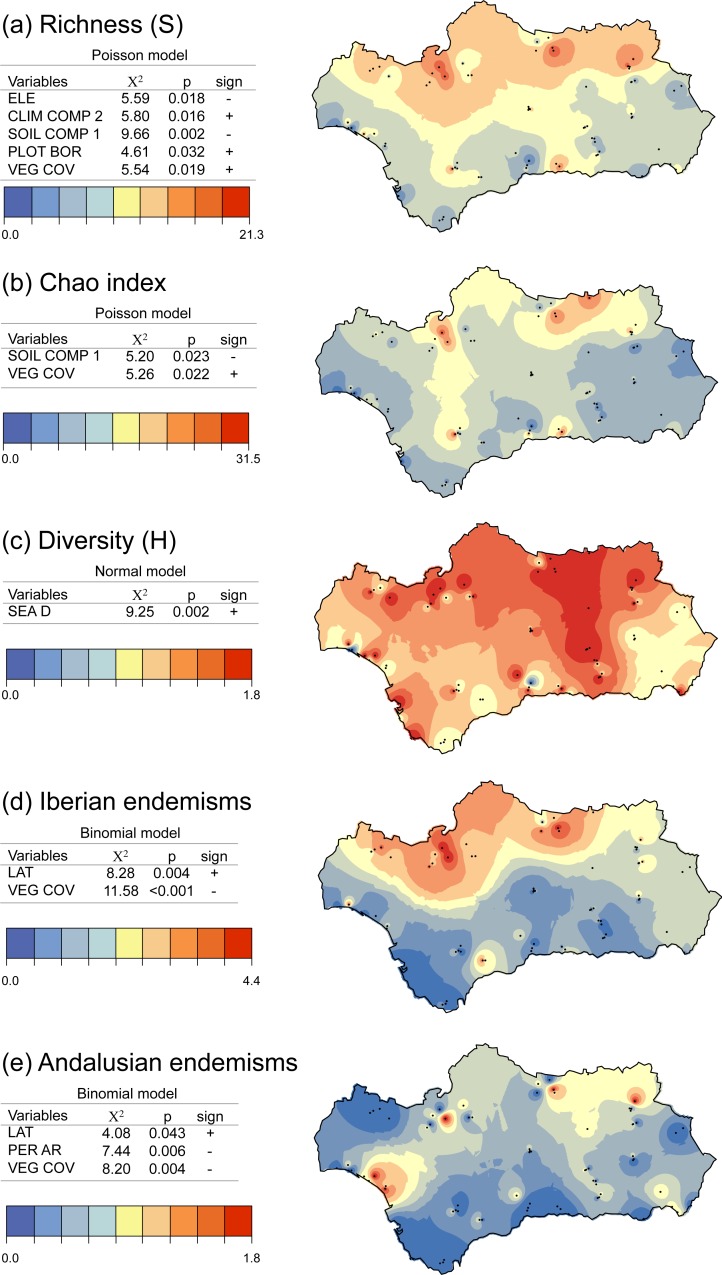
Distribution of conservation indices: (A) species richness, (B) Chao estimated species richness, (C) Shannon diversity, and the proportion of (D) Iberian and (E) Andalusian endemics in protected areas and the surrounding land. Maps were constructed by interpolation of the plots data. In each case, we present the final model with the variables that had a significant effect and the associated statistics (X^2^ and *p*), the sign of the effect (positive or negative), and the distribution of errors used (normal, binomial, or Poisson).

Observed species richness (*S*) decreased significantly with ELEVATION (Fig. 4A). It was also negatively correlated with SOILCOMP1 and positively correlated with CLIMCOMP2 (Fig. 4A). This result means that more species were found on soils with higher concentrations of fine sand, organic matter, and potassium and in areas with more Mediterranean climates (that is, where the annual temperature range is broader, the maximum temperature of the warmest month is higher, and mean annual precipitation is lower). Richness (*S*) was also significantly higher in plots with more vegetation cover (VEGCOV) and in plots further away from the PA border (PLOTBOR).

Chao estimated richness was negatively correlated with SOILCOMP1 and positively correlated with VEGCOV (Fig. 4B), while the value of Shannon’s *H* index was lower in plots closer to the sea (SEAD; Fig. 4C).

Proportions of Iberian and Andalusian endemics (*Ei* and *Ea*) increased significantly with increasing LAT and decreasing VEGCOV (Figs. 4D and 4E). In particular, *Cataglyphis* species frequently occurred inland and in open habitats. Vegetation cover ranged from 0 to 94% and had a mean value (SD) of 25.9% (27.6). However, mean VEGCOV was lower (20.2%) in plots where Iberian *Cataglyphis* endemics occurred (*N* = 25; *C. iberica* and *C. hispanica*) and in plots with Andalusian *Cataglyphis* endemics (VEGCOV = 17.5%; *N* = 17; *C floricola*, *C. tartessica*, *C. rosenhaueri*, *C. velox*, and *C. humeya*). Furthermore, Ea was also significantly and negatively correlated with PERAR (Fig. 4E). On average (SD), the vegetation patches around the plots were 180 (264) ha in size and had a perimeter-to-area ratio of 101.26 (89.6) m/ha. There were more Andalusian endemics in vegetation patches with lower perimeter-to-area ratios; these were patches with a more circular form, which indicated that they were less fragmented.

**Figure 5:**
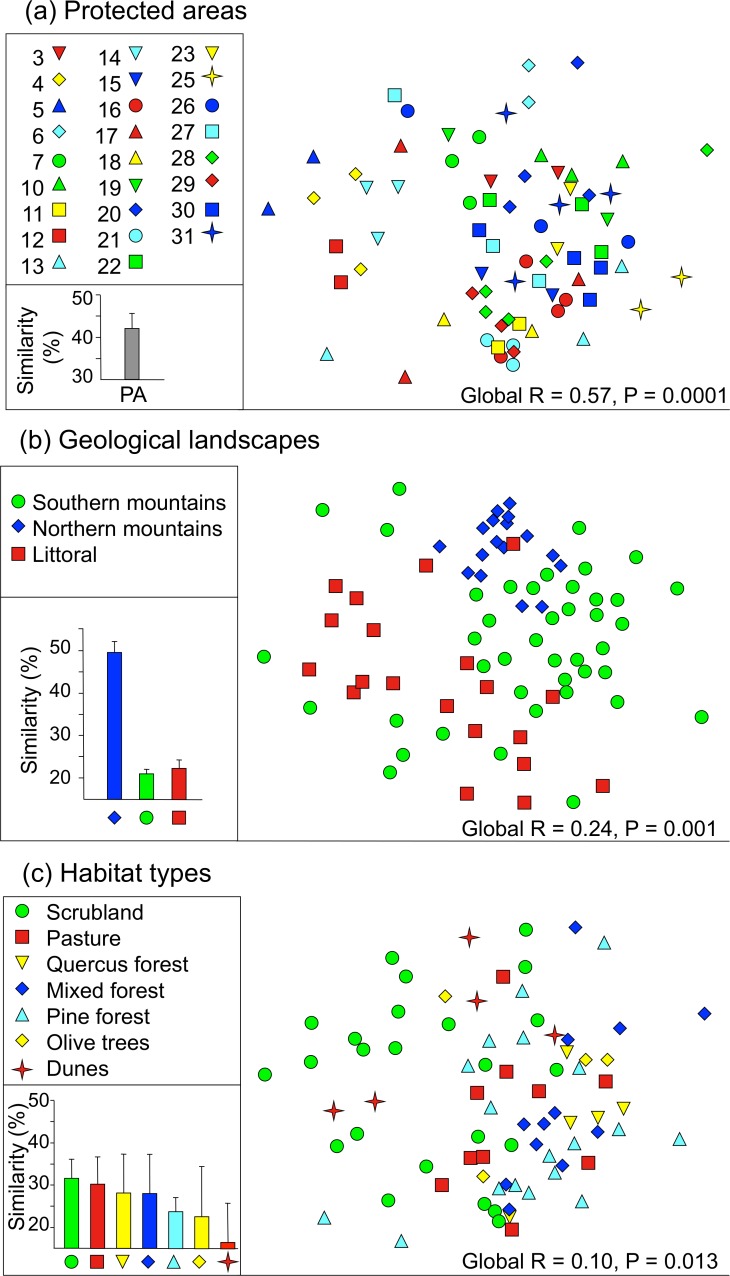
Ordination of ant community similarity. (A) Protected areas, (B) geological landscape types, and (C) habitat types. The global *R* and the associated *p* value indicate the magnitude of the difference among categories (0: no difference, 1: completely different). In (A), each protected area code corresponds to the codes provided in Fig. 1A. Mean (SD) Bray–Curtis similarities between categories are shown in each case except in (A), where mean similarities are shown for pairs found in the same protected area (PA).

None of our diversity indices correlated with vegetation type (VEGTYP), geological landscape type (GEOLAND), or level of anthropogenic disturbance (ANTDI).

### Ant community homogeneity among protected areas

Ant communities were significantly more similar within than among PAs (global *R* = 0.570, *p* < 0.001). Hence, two plots located in the same PA shared, on average, 42% of their species (Fig. 5A). Ant communities within the same type of geological landscape were also similar, although global *R* was lower but significant (global *R* = 0.236, *p* = 0.001). In particular, pairs of plots characterized by the northern mountain chain landscape type shared 49.6% of their species (Fig. 5B). In contrast, within the southern mountain chain and littoral groups, plots were much less similar: they shared, on average, 21% and 22.2% of their species, respectively. Plots with similar vegetation types also had similar ant communities; they shared up to 35% of their species (Fig. 5C); global *R* = 0.098, *p* = 0.013). However, anthropogenic disturbance did not influence ant community similarity (global *R* =  − 0.03, *p* = 0.873).

## Discussion

PAs are a central part of biodiversity conservation efforts and it is vital to determine if they maintain the biodiversity within their boundaries, buffering it from threatening processes. Here, we assessed the effectiveness of the PA network in southern Spain by analysing the anthropogenic factors that currently have the potential to affect biodiversity patterns. In fact, the selection of PAs in the Iberian Peninsula has already been shown to be effective in representing existing natural biodiversity (Araújo, 1999; Araújo, Lobo & Moreno, 2007; Gaston et al., 2008). Our approach thus allows the identification of significant shortfalls in the conservation performance of already established PAs. Using an extensive survey of ant communities across Andalusia (southern Spain), we found that conservation managers should take measures to reduce fragmentation within PAs and take into account the striking differential effects of vegetation cover in their efforts to preserve biodiversity.

### The varying effects of vegetation cover

A major result of our extensive survey is that vegetation cover had opposing effects on the proportion of endemic species and species richness. Plots with less vegetation cover had relatively higher proportions of endemic species (which are mostly hot-climate specialists), but they were also species poor. Our results concur with others in underscoring that richness and endemism do not always coincide spatially (Reid, 1998; Bonn, Rodriguez & Gaston, 2002). Reid (1998) shows how endemic birds species do not satisfactory represent all bird species, particularly not in peak abundance locations. Specifically, Stirnemann et al. (2015) shows how vegetation heterogeneity can affect differently bird richness and functional groups of birds. In Mediterranean ecosystems, vegetation cover protects animal communities from extreme temperatures and low humidity. It shapes competitive interactions among species and is an important structuring force in biotic communities (Retana & Cerdá, 2000). Vegetation cover could constitute a ‘keystone structure’ for ants, as per Tews et al. (2004), as its presence determines ant species diversity and abundance (Bestelmeyer, 2005). Many of the endemic ant species on our plots are thermophilic (10 out of 18): they live in open habitats, either in the mountains or in sparse scrublands (Cerdá, Retana & Manzaneda, 1998). This striking differential effect of vegetation cover on richness and endemism strongly suggests that the management of vegetation cover should be carefully implemented in each ecosystem under consideration. Vegetation cover is intensively managed in protected Mediterranean areas: forests, woodlands, and scrublands suffer from certain management practices (EC, 2006; Lindenmayer et al., 2008). For example, forest clearing and logging may create the conditions necessary to maintain rare endemic species adapted to hot environments, but these practices will inevitably reduce overall ant diversity (Del Toro et al., 2013).

### Human impact in protected areas: biotic homogenization

That environmental gradients have an important influence on variation in community composition is well known (Kraft et al., 2011; Arnan, Cerdá & Retana, 2014). Because environmental variables are spatially structured, they induce spatial dependence in community assemblages (Legendre, Bocard & Peres-Neto, 2005). In this study we found that ant communities were more similar to their near neighbours than to communities farther away, which matches previous studies on ants (e.g., Paknia & Pfeiffer, 2011). To our knowledge, this is the first study to examine variation in compositional similarity among animal communities within a PAs network. If each PA maintains distinct ant community assemblages (as we found here), then the general goal of preserving regional biodiversity is met (Legendre, Bocard & Peres-Neto, 2005).

The process of biotic homogenization has been shown to be dependent on land uses and human disturbance (Florencio et al., 2013; Florencio et al., 2015; Solar et al., 2015). Here we found that human disturbance did not influence ant community similarity. However, ant communities of the same geological landscape type were more similar than communities of the same habitat type. This pattern is probably linked to the species pool and regional processes, which affect speciation and richness. In fact, northern PAs are characterized by more homogeneous climate, geology and vegetation types—mostly mixed forests—while southern PAs are more heterogeneous and comprise extreme habitats, such as high mountains, deserts, heathlands, and coastal areas. Our results suggest that management should prioritize the conservation of the whole landscape; such a management strategy would be in line with strategies used for landscape conservation (Lindenmayer et al., 2008).

### Human impacts on protected areas: fragmentation

The proximity and quantity of human settlements and crops have been shown to be factors that greatly reduce biodiversity in highly urbanized areas (e.g., Sanford, Manley & Murphy, 2009; Spear et al., 2013). In a recent study, Gibb et al. (2015) showed that ant richness decreased in highly disturbed and transformed areas worldwide. However, our index of anthropogenic disturbance, which accounted for the presence of garbage, trails, roads, human construction and evidence of soil manipulation, showed no relationship with the diversity indices or with biotic homogenization. This finding implies that PAs in southern Spain effectively maintain (ant) biodiversity or that the influence of anthropogenic factors is limited.

However, species richness was higher in plots at a greater distance from the PA’s borders and there were more endemics in less fragmented patches, which indicates that ant communities were better preserved in areas with less human interference. Studies using different taxa have recently showed similar results; for example, bird diversity has been showed to respond to distance to PA boundary (Ikin et al., 2014; Wood et al., 2015; Rayner et al., 2015). More generally, several authors have recently stressed the importance of fragmentation, landscape configuration and the maintenance of native biodiversity on lands adjacent to protected areas (Crist, Wilmer & Aplet, 2005; Pryke & Samways, 2012; Villard & Metzger, 2014).

Legendre, Bocard & Peres-Neto (2005) highlighted the critical importance of the existence of patches with homogeneous vegetation and of sufficient size in maintaining endemic species. Similarly, ant species richness has been shown to increase as vegetation fragment size increases in Mediterranean Californian canyons (Suarez, Bolger & Case, 1998), in forest remnants in Brazil (Carvalho & Vasconcelos, 1999), and in fragments of natural vegetation in Australia (Gibb & Hochuli, 2002; Debuse, King & House, 2007). The fact that we identified boundaries of patches as an abrupt change of habitat type or the presence of linear gaps (e.g., trails, roads) suggests that a reduction of linear gaps within PAs should be crucial in the management of these areas. Roads and trails networks optimization has been shown to have positive effects at ecosystem level (D’Amico et al., 2016).

## Conclusion

Given the response of ant communities in PAs to certain anthropogenic factors, we suggest the following to further increase PA’s efficiency for biodiversity conservation: (1) the effect of vegetation management tools (e.g., clearing and logging) should be carefully studied in each landscape type, as richness and endemism could demonstrate contrasting responses; (2) managers should prioritize landscape conservation instead of focusing on specific habitat types; and (3) management programs should focus on reducing fragmentation within PAs, specifically roads and trails that fragment habitat patches.

Although it has some limitation, sampling ant communities using pitfall traps is a standard and recognized method that allows comparisons between sites and even between studies. This sampling that was done nearly one decade ago also provides a baseline for future re-sampling in order to detect the possible changes in ant communities driven by global change. Although the use of multiple and complementary surrogates to assess the state of biodiversity should be chosen when possible (Sattler et al., 2013; Lentini & Wintle, 2015), we think our analyses are valid because some of our results lead to similar conclusions as studies on other species (e.g., Crist, Wilmer & Aplet, 2005; Pryke & Samways, 2012; Villard & Metzger, 2014; Rayner et al., 2015; Wood et al., 2015). Revealing the patterns that impact the efficiency of PAs networks for biodiversity conservation, our results can inform management decisions and influence the prioritization of conservation efforts. This is especially important given that the future PAs networks will likely face fragmentation problems due to the increase in the proportion of smaller PAs (Maiorano et al., 2015; Meyer, Beard & Cronan, 2015) and urban expansion around PAs (Bonet-García et al., 2015; Martinuzzi et al., 2015; Wood et al., 2015).

##  Supplemental Information

10.7717/peerj.2773/supp-1 Supplemental Information 1 Description of the sites sampled in the Andalusia network of protected areas (PAs)

## Additional Information and Declarations

### Field Study Permissions

The following information was supplied relating to field study approvals (i.e., approving body and any reference numbers): Regional Government of Andalusia (Consejería de Medio Ambiente) issued permits for the fieldwork in Protected Areas.

### Data Availability

The following information was supplied regarding data availability: Research Gate; DOI: 10.13140/RG.2.2.16395.49441; https://www.researchgate.net/publication/309513177_.
