# Supplementary material for: Anthropogenic impacts in protected areas: assessing the efficiency of conservation efforts using Mediterranean ant communities
Source: PeerJ. 2016 Dec 14;4:e2773. doi: 10.7717/peerj.2773 (PMC5160926; doi:10.7717/peerj.2773)
Supplement: Supplemental Information 1 [file peerj-04-2773-s001.pdf]

Table S1: Description of the sites sampled in the Andalusia network of protected areas (PAs)

PA # [a] and name	N[b]	S[b]	E[b]	Landscape [c]	Vegetation [d]	Altitude[e]
1. Acantilados de Maro-Cerro Gordo	1	12	2	L	Sc	10
2. Bahía de Cádiz	1	4	0	L	Du	0
3. Breña y Marismas de Barbate	2	15	0	L	Pi, Du	38
4. Cabo de Gata-Níjar	3	18	3	L	Sc	20
5. Desierto de Tabernas	2	11	3	S	Sc	0
6. Doñana, Parque Nacional	2	13	4	L	Sc	20
7. Doñana, Parque Natural	3	21	4	L	Sc, Pi, Du	58
8. Dunas de San Antón	1	10	0	L	Du	21
9. Enebrales de Punta Umbría	1	9	0	L	Pi	13
10. Los Alcornocales	3	14	1	S	Sc, Mi	163
11. Marismas del Odiel	2	16	5	L	Sc, Pi	4
12. Marismas Río Piedras y Flecha del Rompido	2	5	0	L	Sc, Du	0
13. Montes de Málaga	3	14	1	S	Pi	757
14. Punta Entinas-Sabinar	3	15	1	L	Sc	4
15. Sierra de Alhama, Tejeda y Almijara	2	19	2	S	Sc, Pi	709
16. Sierra de Aracena y Picos de Aroche	4	28	4	N	Sc, Pi, Mi	621
17. Sierra de Baza	3	24	5	S	Sc, Pi	1761
18. Sierra de Cardeña y Montoro	2	20	5	N	Pa, Pi	655
19. Sierra de Castril	2	14	5	S	Ol, Pa	1089
20. Sierra de Grazalema	4	24	3	S	Pi, Mi, Ol, Pa	849
21. Sierra de Hornachuelos	3	18	4	N	Sc, Mi, Pa	282
22. Sierra de Huétor	3	25	5	S	Sc, Mi, Co	1378
23. Sierra de las Nieves	2	16	5	S	Mi, Co	1107
24. Sierra Mágina	1	10	3	S	Sc	1972
25. Sierra María-Los Vélez	1	9	3	S	Pi	1245
26. Sierra Nevada, Parque Nacional	3	19	4	S	Sc, Mi, Co	1753
27. Sierra Nevada, Parque Natural	3	19	3	S	Sc, Pi, Mi	1741
28. Sierra Norte de Sevilla	5	35	10	N	Sc, Mi, Ol, Pa	336
29. Sierras de Andújar	3	31	8	N	Pa, Pi, Co	515
30. Sierras de Cazorla, Segura y las Villas	4	32	5	S	Pa, Mi, Co	1098
31. Sierras Subbéticas	4	27	4	S	Pa, Ol, Sc	872
32. Torcal de Antequera	1	9	3	S	Pa	1224

[a] PA # corresponds to the codes provided in Figure 1a.

[b] N refers to the number of plots, S to the observed richness, and E to the total number of endemic species (Ei+Ea) in each PA.

[c] Landscape is abbreviated as follows: northern mountain chain (N), southern mountain chain (S), and littoral (L).

[d] Vegetation type is abbreviated as follows: scrubland (Sc), pine forest (Pi), mixed forest (Mi), cork oak forest (Co), olive trees (Ol), dune (Du), and pasture (Pa).

[e] Altitude refers to the mean altitude of the plots (m).

Table S2: Factor coordinates and cumulative eigenvalues for the climate and soil composition variables.

**Climate variables**	Factor 1	Factor 2
Annual mean temperature	**-0.94**	0.06
Maximum temperature of the warmest month	0.11	**0.58**
Minimum temperature of the coldest month	**-0.98**	-0.13
Annual range of temperature	**0.83**	0.41
Annual precipitation	**0.58**	**-0.74**
Precipitation in the wettest month	0.27	**-0.87**
Precipitation in the driest month	**0.84**	0.21
Cumulative eigenvalue %	52.4	79.4

**Soil composition variables**	Factor 1	Factor 2	Factor 3
Organic material	**-0.76**	-0.09	-0.37
Rocky sand	**0.95**	-0.13	-0.19
Fine sand	-0.49	**0.70**	0.08
Clay	**-0.93**	0.16	0.11
K – Potassium	**-0.76**	-0.43	0.02
P – Phosphorus	-0.34	**-0.65**	-0.26
pH	-0.01	-0.31	**0.93**
Cumulative eigenvalue %	46.8	64.7	80.4

Table S3 - List of the ant species collected indicating their abundances in the pitfall, occurrence in the sampling points (total number of sampling points: 607) and incidence in the plots (total number of plots: 79).

	Species	Pitfall catches	Sampling points	Plots
1	*Aphaenogaster dulcineae*	7	3	2
2	*Aphaenogaster gibbosa*	2659	152	32
3	*Aphaenogaster iberica*	3222	197	37
4	*Aphaenogaster senilis*	3096	165	31
5	*Camponotus amaurus*	77	16	4
6	*Camponotus cruentatus*	1388	75	22
7	*Camponotus foreli*	112	23	9
8	*Camponotus lateralis*	583	38	19
9	*Camponotus piceus*	242	31	10
10	*Camponotus pilicornis*	542	124	37
11	*Camponotus ruber*	8	3	2
12	*Camponotus sylvaticus*	125	38	17
13	*Cardiocondyla batesii*	24	4	1
14	*Cataglyphis floricola*	214	16	4
15	*Cataglyphis tartessica*	999	19	3
16	*Cataglyphis hispanica*	2522	109	16
17	*Cataglyphis humeya*	334	8	1
18	*Cataglyphis iberica*	1082	62	9
19	*Cataglyphis rosenhaueri*	541	45	12
20	*Cataglyphis velox*	1437	63	11
21	*Crematogaster auberti*	4435	179	46
22	*Crematogaster scutellaris*	1032	47	21
23	*Crematogaster sordidula*	129	20	12
24	*Formica decipiens*	948	51	15
25	*Formica pyrenaea*	1776	8	2
26	*Formica rufibarbis*	1544	20	5
27	*Formica subrufa*	9048	102	24
28	*Goniomma baeticum*	2	2	2
29	*Goniomma blanci*	19	12	6
30	*Goniomma hispanicum*	5	3	3
31	*Lasius brunneus*	2	2	2
32	*Lasius cinereus*	204	18	3
33	*Lasius grandis*	18	2	1
34	*Lasius myops*	9	4	2
35	*Lasius niger*	3053	36	11
36	*Lepisiota frauenfeldi*	562	12	3
37	*Leptothorax angustulus*	347	50	21
38	*Leptothorax pardoi*	616	45	25
39	*Leptothorax racovitzai*	1873	108	37
40	*Leptothorax sp.*	26	9	5
41	*Leptothorax unifasciatus*	74	17	6
42	*Linepithema humile*	8081	24	4
43	*Messor barbarus*	3079	67	22
44	*Messor bouvieri*	2598	26	12
45	*Messor capitatus*	4124	16	6
46	*Messor hispanicus*	1505	19	10
47	*Messor lusitanicus*	79	20	9
48	*Messor marocanus*	102	2	2
49	*Messor structor*	277	25	7
50	*Monomorium algiricum*	4	2	2
51	*Monomorium subopacum*	8692	73	14
52	*Myrmica aloba*	1606	9	4
53	*Oxyopomyrmex saulcyi*	17	7	5
54	*Pheidole pallidula*	10550	166	41
55	*Plagiolepis pygmaea*	2252	150	43
56	*Plagiolepis schmitzi*	293	51	24
57	*Proformica longiseta*	1083	23	3
58	*Diplorhoptrum sp.1*	64	24	11
59	*Strongylognathus testaceus*	2	1	1
60	*Tapinoma erraticum*	499	24	10
61	*Tapinoma nigerrimum*	7913	130	36
62	*Tapinoma simrothi*	6	2	1
63	*Tapinoma sp.1*	1	1	1
64	*Temnothorax fuentei*	23	10	2
65	*Temnothorax prope naeviventris*	1	1	1
66	*Temnothorax recedens*	89	1	1
67	*Temnothorax specularis*	131	3	3
68	*Tetramorium caespitum*	152	38	18
69	*Tetramorium impurum*	1442	84	34
70	*Tetramorium semilaeve*	538	107	45
